# The effectiveness of a proposed program to enhance social and emotional skills on developing psychological well-being (good condition) in UNRWA schools: Jalazone Basic Schools as a model

**DOI:** 10.3389/fpsyt.2026.1761736

**Published:** 2026-07-24

**Authors:** Maurice Backleh, Iyad Yousef, Abdallah Bsharat, Refa’ Al-Ramahi, Fatima Hamad, Sana Liftawi, Bihan Qaimari

**Affiliations:** 1 Department of Social and Behavioral Sciences – Psychology Program, Birzeit University, Ramallah, Palestine; 2 Department of Physical Education, College of Education, Birzeit University, Ramallah, Palestine; 3 Department of Curriculum and Instruction, Faculty of Education, Birzeit University, Ramallah, Palestine; 4 Kutayba Yousef Alghanim Chair in Education, Birzeit University, Ramallah, Palestine; 5 Department of Inclusive and Special Education, Faculty of Education, Birzeit University, Ramallah, Palestine

**Keywords:** emotional skills, good condition, Jalazone Basic Schools, psychological well-being, social skills, UNRWA schools

## Abstract

**Objective:**

This study aimed to examine the effectiveness of a proposed program to enhance social and emotional skills in developing mental health among seventh-grade students at the Jalazone Basic School for Boys and Girls, run by the United Nations Relief and Works Agency for Palestine Refugees (UNRWA).

**Methods:**

To achieve the study’s objective, a mixed-methods approach, including quasi-experimental, quantitative, and qualitative research, was employed. The researchers used two research tools: The Psychological Well-being Scale and a semi-structured interview to collect quantitative and qualitative data. The results will be analyzed using descriptive and inferential statistics to answer the study questions and determine the statistical significance of any differences between the male and female groups. Content analysis will also be conducted on the qualitative data collected through the semi-structured interview tool. This study implemented a program that enhances social and emotional learning (SET) in a seventh-grade class at a boys’ school and a seventh-grade class at a girls’ school.

**Results:**

The pre-test findings confirmed the groups’ baseline equivalency by showing no statistically significant differences between the experimental and control groups on the majority of social-emotional skills and psychological well-being measures. The pre-test results confirmed the original baseline equivalency between male students in both groups, showing no statistically significant differences between the experimental and control groups across the majority of social-emotional skills and psychological well-being categories. According to the findings of the Two-Way ANCOVA analysis of variance, the suggested training regimen resulted in statistically significant group differences, with the group factor having the greatest influence on all dependent variables. Finally, through this implemented program, students learn how to resolve problems and conflicts and manage their own emotions as well as the emotions of others.

**Conclusion:**

This research contributes to the existing literature by developing psychological well-being and creative methods for implementing interventions that increase the influence and reach of evidence-based practice across various educational systems and school settings, which may result from identifying a shared set of evidence-based techniques to improve adolescents’ social and emotional skills. This study fills a knowledge gap regarding gender differences in emotional and social skills and their significance in promoting psychological well-being among UNRWA students in the Jalazone Palestinian refugee camp. Addressing these variations among this student population and understanding their underlying reasons will aid in developing effective, culturally relevant, and individualized treatments for each group.

## 1 Introduction

Students in many marginalized areas, and the Jalazone camp in particular, suffer from many problems related to their psychological health and learning due to the housing conditions inside the camp, which suffers from weak infrastructure, overcrowding, poverty, and other difficult living conditions. On the one hand, and on the other hand, Jalazone camp in particular, due to its proximity to the so-called Beit El settlement, which was built on the lands of the city of Al-Bireh and the villages of Ein Yabroud and Dura Al-Qara, and has directly faced the camp since 1978, and the daily provocations of the occupation army and the settlements or colonists against school students, causing them suffering, anxiety, distress, and other things.

The suffering of the camp’s residents has been exacerbated by the proximity of the settlement, martyrdom of their children, injuries among school students, and closure of the road adjacent to the camp for many years before the occupation opened it and then closed it again from time to time (1, 2). Therefore, it was necessary to intervene to save the difficult psychological situation that camp residents suffer from.

According to a 2018 report by B’Tselem, the Israeli Information Center for Human Rights in the Occupied Territories, the Jalazone camp is located north of Ramallah. The camp was established in 1949 to provide temporary housing for Palestinians who became refugees during the 1948 war, and whom Israel refused to allow to return to their homes after the war ended. The camp’s residents originate from 36 villages in the Lod, Ramle, Haifa, and Western Hebron areas. According to records from the United Nations Relief and Works Agency for Palestine Refugees in the Near East (UNRWA), the current population of the Jalazone camp, seven decades after its establishment, is 14,579, including 5,151 children and youth under the age of 18 (3).

At the main entrance to the camp, outside its official boundaries, the United Nations Relief and Works Agency for Palestine Refugees (UNRWA) established two schools on the land of Jifna village, serving approximately 2,000 students. The camp’s residents suffer from severe overcrowding and have exhausted all available construction opportunities within its boundaries. The lands outside the camp, where the new neighborhoods and schools were built, were classified as Area C under the Oslo Accords. Israel reserved the planning, construction, and development powers in these areas. Camp residents who wish to build or expand their homes in these areas are forced to contend with Israel’s sweeping refusal to grant building permits in Area C. The Civil Administration issues demolition orders for homes built by their owners—without a building permit. From the time the demolition order was issued, whether or not it was implemented, the specter of demolition loomed over the residents (4).

Israel treats camp residents—even in their own homes—as unwelcome guests and shuns any obligation toward them. It forces them to live in extreme overcrowding within the camp’s boundaries—drawn 70 years ago—and prohibits new construction outside the camp. Meanwhile, Israel continues to use the lands of neighboring Palestinian towns for its own purposes, including the expansion of settlements, consistently prioritizing the interests of settlers over those of Palestinians, in complete disregard of their rights. This is how the occupation regime operates in the Jalazone refugee camp and throughout the rest of the West Bank (5).

The Watan News Agency reported on May 15, 2018, the suffering of the residents and children of the Jalazone camp. It reported severe overcrowding in all classrooms in UNRWA schools, as well as in clinics and medical centers. There is a shortage of medicines and termination of the food aid program. There is a neglect of hygiene and infrastructure. The agency is now liquidating its employment program, which benefits 70 families per month in the Jalazone camp alone.

The recent period, especially during COVID-19 and the use of distance learning, has witnessed widespread mental health problems among children and adolescents. This has manifested in various forms of aggressive behavior, violence, depression, loneliness, anxiety, and other disruptive behaviors, in addition to many other, less dramatic forms that fall under the umbrella of mental health problems, which inevitably impact students’ learning and motivation to learn and achieve. Even before COVID-19, children and adolescents have borne the brunt of mental health problems in the absence of any specific investments to intervene or address them.

COVID-19 has had significant health, economic, social, and psychological impacts worldwide, paralyzing and disrupting people’s lives. Globally, it is estimated that more than one in seven adolescents aged 10–19 years have been diagnosed with a mental health disorder. Approximately 46,000 adolescents die by suicide each year, making it one of the top five causes of death among this age group. Meanwhile, wide gaps remain between mental health needs and funding.

### 1.1 Problem statement

Primary education is one of the most important educational stages in an individual’s life, as it is an educational stage in itself, working to prepare students psychologically, mentally, motorically, and socially for education and social life. In light of the rapid changes that the world in general and Palestine in particular have experienced during the Corona pandemic, which affected all aspects of life, including education and health, many countries adopted preventive measures to control the spread of the virus. These measures included restrictions on people’s freedom of movement and preventing their movement, such as quarantine and social isolation, which resulted in several negative physical and psychological consequences, such as increased levels of anxiety, stress, fear, and the emergence of symptoms of depression, which greatly affected university and school students (6–8).

Many psychological studies, such as the study by Prajapati, Sharma, and Sharma (9), have confirmed the importance of social and emotional life skills in students’ lives, as they are an urgent necessity in the basic education stage and play an important role in promoting psychological, mental, and physical health, improving personal and social relationships and self-esteem in students, and reducing social and behavioral problems that students are exposed to. The World Health Organization report (10) emphasized the necessity of teaching individuals’ life skills to develop their psychological and social abilities and positive adaptation that enables them to deal effectively with the demands and challenges of daily life.

In Egypt, Abdel Latif (11) conducted a study aimed at identifying the level of psychological stress and resilience among third-grade primary school students in Cairo, and identifying the differences between the sexes and which of them is affected by it. The sample consisted of (90) male and female primary school students, and the basic sample consisted of (100) male and female third-grade primary school students.

The researcher used the psychological stress and psychological resilience scales prepared by the researcher. The results showed the existence of a statistically significant direct relationship between the total score of psychological stress and the total score of psychological resilience among primary school students, and the existence of a statistically significant direct relationship between personal psychological stress and the mental and social aspects and the total score of psychological resilience among primary school students, except for the emotional aspect. There was a statistically significant direct relationship between health psychological stress and the emotional and mental aspects and the total score of psychological resilience among primary school students, except for the social aspect at 0.05, and the existence of a statistically significant direct relationship at the 0.01 level between social psychological stress and the emotional aspect of resilience. Psychological and overall degree of psychological flexibility among primary school students, except for the mental and social aspects, and the absence of statistically significant differences between the average ranks of primary school students in psychological flexibility and its sub-dimensions: emotional, social, and mental, attributed to gender (males and females).

Through the researchers’ experience in the educational and psychological fields, their visits to many public and private schools and UNRWA schools, and their interviews with some teachers, they noticed that there is a weakness in students’ possession of social and emotional skills that enable them to live with better social and psychological health, increase their achievement, motivation, and focus toward learning, and reduce problems and school violence among students, with a clear gap and disparity between students in various life skills such as time management, emotional control, social competence, and self-confidence. From here, the problem of the study crystallized for the researchers in the following study question: What is the effectiveness of the proposed program in enhancing social and emotional skills in developing psychological well-being (good condition) in UNRWA schools?

### 1.2 Research objectives

The objectives of this study are as follows:

1. Differences in psychological well-being before and after implementing the social and emotional skills enhancement program.
2. The differences between males and females in psychological well-being.
3. How the program enhances students’ psychological well-being skills according to the perspectives of teachers and counselors.
4. How the program enhances students’ psychological well-being skills, according to the perspectives of their parents.

### 1.3 Research questions

What is the effectiveness of the proposed program in enhancing social and emotional skills to develop psychological well-being (well-being) in UNRWA schools? The following sub-questions arise from the main question:

1. Is there a difference in students’ average performance on the psychological well-being scale before and after the program’s implementation?
2. Is there a difference in students’ average performance on the psychological well-being scale attributable to gender.
3. How did the program enhance students’ psychological well-being skills according to the perspectives of teachers and counselors?
4. How did the program enhance students’ psychological well-being skills, according to the perspectives of the students’ parents?

### 1.4 Research hypotheses

Based on the main study questions above, the following three hypotheses emerge:

1. There were no statistically significant differences between the mean scores of female students in the experimental and control groups in the pre- and post-test measurements on the social-emotional skills and psychological well-being scale.
2. There were no statistically significant differences between the mean scores of male students in the experimental and control groups in the pre- and post-test measurements on the social-emotional skills and psychological well-being scale.
3. There were no statistically significant differences in the post-test means of social-emotional skills and psychological well-being variables (COMM, LIST, PROB, TEAM, SELF, EMOT, and WELL-BEING) attributable to group (experimental vs. control), gender (male vs. female), or their interaction (12).

### 1.5 Study importance

The importance of this research lies in the fact that the Social and Emotional Training (SET) program, which will be implemented in the Jalazone Boys’ and Girls’ Elementary Schools, focuses primarily on preventing mental health problems or deterioration among children and students in these two schools. The goal is to equip them with a variety of social and emotional skills and ensure that they are practiced in their daily lives. This is based on the premise that interventions should focus on risks and protection rather than on abnormal behaviors.

Palestinian education, like other aspects of life, has been affected by the political conditions. In contrast, politics are not far from the schoolyard in the occupied territories and conflict zones. Schools have become a center of conflict between Zionist soldiers and Palestinian youths. Students have become the fuel for this conflict, throwing stones and being subjected to arrest, beatings, martyrdom, and school closure. This is also the case for teachers, especially in the event of civil unrest. Children are forced to mature politically as a result of the pressures of their environment, even though this burdens them with anxiety and tension. Students are exposed to numerous racist and inhumane practices in schools. Palestinian refugee camps in the occupied West Bank, and Jalazone camp in particular, experience ongoing confrontations and attacks by occupying forces, resulting in hundreds of injuries, thousands of disabilities, hundreds of arrests, and dozens of deaths. All of this has hurt the mental health and well-being of school students in India. This study is important because it focused on seventh-grade students, who are beginning adolescence. It is important to work with students to improve their social and emotional life skills, which will enhance their psychological wellbeing.

The program primarily focuses on preventing mental health problems or deterioration among children or students in these two schools, with the goal of providing them with a variety of social and emotional skills and ensuring their application in their daily lives. Several modifications were made to the model to suit the Palestinian context in collaboration with several researchers. The Palestinian Ministry of Education then studied it and adopted it for implementation in several schools in several governorates. The interventions to be implemented focus on risks and protection rather than on abnormal behaviors themselves. The Social and Emotional Training (SET) program is a basic program that provides students with specific skills for social interaction during childhood and adulthood.

The Social Emotional Training (SET) program is an essential program that equips students with specific skills for social interaction with others during childhood and adulthood. Students learn how to solve problems and conflicts, deal with their own emotions, and deal with the emotions of others. They also learn how to manage their lives in a healthier manner. Therefore, this study is expected to benefit teachers, counselors, and the Psychological and Educational Counseling Department at UNRWA, as it will provide them with insights into how to enhance students’ social skills and emotional expression, which may improve their psychological well-being.

Identifying a common set of evidence-based strategies to enhance adolescents’ social and emotional skills could lead to the development of psychological well-being and innovative approaches to delivering interventions that expand the impact and reach of evidence-based practice across diverse educational systems and school settings. This research contributes to the existing literature by developing psychological well-being and creative methods for implementing interventions that increase the influence and reach of evidence-based practice across various educational systems and school settings, which may result from identifying a shared set of evidence-based techniques to improve adolescents’ social and emotional skills.

## 2 Literature review

### 2.1 Trauma, conflict exposure, and mental health

According to early results from an international survey conducted by UNICEF and Gallup, which included children and adults in 21 countries, one in five young people aged 15 to 24 years reported frequently feeling depressed or losing interest in activities (13). The agency stated that as the pandemic entered its third year, the mental health and well-being of children and young people continued to be significantly affected.

Many schools, especially those located in marginalized areas and communities surrounding settlements, face substantial psychological and social challenges owing to prolonged exposure to conflict, instability, and the lingering effects of the COVID-19 pandemic. In a study assessing the impact of COVID-19 on mental health and quality of life in the Middle East and North Africa (MENA) region, AlDhaheri et al. (14) surveyed 6,142 adults from 18 countries. The findings revealed that most participants (45%–62%) experienced fear, anxiety, and helplessness because of the pandemic. Furthermore, 30.9% reported severe psychological distress, while more than 40% reported increased stress related to work and financial concerns. Women, North African participants, and individuals aged 26–35 years reported the highest levels of distress.

Similarly, Nathani et al. (8) investigated the relationship between mental well-being and school attendance among Palestinian adolescent refugees attending UNRWA schools in the occupied Palestinian territories, Jordan, Lebanon and Syria. Their findings indicate that adolescents experiencing mental health difficulties are more likely to miss school. Females reported higher levels of loneliness and anxiety, whereas males were more likely to be absent from schools. The authors emphasized the need for additional school- and community-based mental health interventions, particularly those tailored to the needs of female refugee adolescents.

Research conducted among Palestinian children has also demonstrated the adverse effects of traumatic experiences on their psychological functioning. Qouta (15) examined the relationship between traumatic experiences and emotional, cognitive, and behavioral responses among 108 Palestinian children aged 11–12 years in the Gaza Strip. The study found that increased exposure to violence and trauma was associated with greater difficulties in concentration and memory, elevated neurotic symptoms, and diminished cognitive and creative abilities.

Likewise, Hijazi (16) explored the relationship between traumatic experiences, post-traumatic stress disorder, and selected personality traits among children of martyrs of the Al-Aqsa Intifada in Palestine. The sample comprised 176 children aged 9–14 years. The findings indicated no statistically significant differences according to place of residence; however, significant sex differences were observed, with males reporting higher levels of traumatic experiences than females.

Szwarcwald et al. (17) reported that excessive exposure to computer and mobile phone screens negatively affected emotional well-being during the pandemic. Females were more vulnerable to emotional problems than males, while adolescents from lower socioeconomic backgrounds experienced greater social isolation and unhealthy behavior.

Recent research has examined the psychological challenges faced by Palestinian refugee children and adolescents living in conflict and displacement contexts. Wilson et al. (18) reported that Palestinian refugee children are at a greater risk of mental health disorders and psychosomatic symptoms than children living in non-conflict settings. This study also highlighted the importance of supportive relationships, educational engagement, and social participation in promoting resilience.

Similarly, Al Issa (19) found that Palestinian refugee children living in camps in Bethlehem experienced multiple forms of trauma associated with conflict and displacement. The findings emphasize the role of self-efficacy across academic, emotional, and social domains as an important protective factor that may enhance resilience and psychological adaptation.

Douglas et al. (20) conducted a meta-analysis and demonstrated that children and adults exposed to displacement, armed conflict, and traumatic events are at an increased risk of anxiety, depression, and other mental health disorders.

Given the psychological challenges experienced by refugee camp students as a result of ongoing conflict-related stressors and difficult living conditions, preventive and developmental interventions are needed to enhance their psychological well-being and promote positive mental health outcomes. Collectively, the findings of previous studies underscore the urgent need for preventive and developmental interventions that strengthen resilience, support mental health, and promote psychological well-being among vulnerable student populations.

### 2.2 Social and emotional learning interventions

Various interventions have been implemented by teachers, counselors, and parents to develop social and life skills and reduce negative behaviors, such as violence, bullying, and social withdrawal (21). However, some interventions have shown limited effectiveness because they do not adequately incorporate cognitive-behavioral components or address broader social and contextual factors affecting their effectiveness (22). Consequently, there is a need for innovative preventive and developmental approaches.

One such approach is the Social and Emotional Training (SET) program, which was developed to enhance students’ psychological well-being, strengthen mental health, and promote the acquisition and application of social and emotional skills in everyday life. The program relies on interactive instructional strategies, including modeling, role-playing, experiential learning, and practical application of learned skills across school, family, and community settings.

Sandell and Kimber (23) conducted a pilot study to investigate students’ responses to a social and emotional skills program implemented in Swedish schools, and The findings revealed variability in student outcomes, suggesting that the program was particularly beneficial for high-risk students, and that the intervention effects differed across participant groups.

Similarly, a quasi-experimental study conducted in Sweden examined the socio-emotional development of students participating in SET programs between 1999 and 2005. Using the How I Feel (HIF) assessment, positive effects on mental health, empathy, and cooperation were found, as well as a negative relationship between bullying and socio-emotional competence (24–27).

Kimber (28) further demonstrated that SET programs contributed to the prevention of mental health problems, reduced school dropout rates, and enhanced school satisfaction. Nevertheless, successful implementation depends on strong community involvement and effective school management.

Additional evidence has highlighted the effectiveness of SET programs when they are implemented by trained teachers. Kimber, Skoog, and Sandell (29) found that participation in SET training improved teachers’ professional performance, classroom climate, communication with parents, and the use of positive instructional strategies.

Likewise, Kimber, Sandell, and Bremberg (30) reported positive outcomes among students participating in SET programs, including improvements in social competence, self-awareness, empathy, and motivation, and reductions in bullying behaviors.

Recent research continues to support the effectiveness of Social and Emotional Learning (SEL) interventions in promoting students’ psychological well-being and social-emotional development. Lee and Yoo (31), in a systematic review, concluded that SEL programs improve adolescents’ emotion regulation, increase happiness, and contribute positively to mental well-being. The authors also emphasized the importance of adapting SEL interventions to the needs of students and educational contexts to maximize their effectiveness.

Similarly, de Mooij et al. (32) found that SEL interventions improved school climate and psychosocial adjustment among school-aged children, highlighting the value of both universal and targeted implementation approaches in this context. Belaire et al. (33) reported significant associations between social-emotional learning, resilience, and psychological well-being among school-aged children, further supporting the importance of SEL-related competencies in promoting positive developmental outcomes.

### 2.3 Gender differences and psychological well-being

Several studies have highlighted the importance of gender in examining psychological well-being and social-emotional development. Nathani et al. (8) found that female refugee adolescents reported higher levels of loneliness and anxiety, whereas males exhibited higher rates of school absenteeism than females. Similarly, Szwarcwald et al. (17) observed that women were more vulnerable to emotional difficulties during the COVID-19 pandemic.

Research has also examined the relationship between psychological well-being and educational outcomes. Berger et al. (34) reported a positive association between emotional well-being and academic achievement. Similarly, Miller et al. (35) found that students with higher levels of well-being demonstrated better academic performance.

In contrast, Van Petegem et al. (22) reported no statistically significant relationship between psychological well-being and academic achievement. Similarly, Elovainio et al. (36) found that higher academic achievement was associated with lower school dissatisfaction levels.

At the university level, the findings remain inconsistent. Sadeghiet et al. (37) found no significant relationship between mental health and academic achievement among university students in Iran. Conversely, Ghamariet et al. (38) reported a significant positive association between mental health and academic achievement.

Despite the growing body of literature on psychological well-being and social-emotional learning, limited research has examined how social and emotional skills interventions influence the psychological well-being of Palestinian refugee adolescents attending UNRWA schools. Furthermore, evidence regarding sex differences in these outcomes remains limited. Therefore, the present study seeks to address this gap by examining the effectiveness of a social and emotional skills enhancement program in improving psychological well-being among students in Jalazone Camp School.

## 3 Design and methodology

### 3.1 Methodology

This study primarily employed a quasi-experimental quantitative design supplemented by qualitative evaluative feedback obtained through semi-structured interviews. The quantitative component utilized a quasi-experimental pretest–posttest control-group design. The study included four groups: two experimental (one male and one female) and two control groups (one male and one female).

Participants in the experimental groups received the Social and Emotional Training (SET) Program, whereas participants in the control groups continued with their regular school activities and did not receive any intervention during the study period.

All groups completed the Psychological Well-Being Scale before the implementation of the program (pre-test) and again after its completion (post-test). The effectiveness of the intervention was evaluated by comparing changes in psychological well-being between the experimental and control groups, while also examining potential gender-related differences. Baseline equivalence between groups was assessed before the intervention, and pre-test scores were controlled for during the statistical analyses.

The qualitative component consisted of semi-structured interviews conducted with participating students after the training program was implemented. Additional evaluation interviews were conducted with educational counselors, teachers, parents, and the school principal to obtain feedback on the implementation and perceived usefulness of the program. These interviews were used as supplementary evaluative information to support the interpretation of the quantitative findings and to assess the acceptability and perceived effectiveness of the intervention. Therefore, the interview data were not subjected to formal thematic analysis or reported as an independent qualitative dataset.

### 3.2 Study sample and population

The study population consisted of seventh-grade students enrolled in UNRWA schools in the Jalazone Palestinian Refugee Camp. A purposive (non-random) sampling approach was used to select the participants. In the boys’ school, three seventh-grade classes were available (Sections A, B, and C). Sections B was selected as the experimental and control groups, respectively. Similarly, in the girls’ school, three seventh-grade classes were available (Sections A, B, and C). Sections B was selected as the experimental and control groups, respectively.

The final study sample consisted of 135 students distributed across four groups: 35 female students in the experimental group, 30 female students in the control group, 36 male students in the experimental group, and 34 male students in the control group. The sample size (N = 135) was considered adequate for detecting meaningful intervention-related differences between groups and for conducting the planned inferential analyses, including t-tests and ANCOVA, while maintaining a sufficient statistical power.

Furthermore, the sample represented the majority of students enrolled in the selected seventh-grade classes, providing an appropriate basis for evaluating the effectiveness of the intervention within the study context.

Participants were eligible for inclusion if they were enrolled in the seventh grade at the selected UNRWA schools, regularly attended school during the study period, and completed both pre- and post-test assessments. Participation was conducted in coordination with the school administration, and all students included in the final analyses completed the intervention and assessment procedures.

Given that the sample was selected purposively from a single refugee camp and educational setting, the findings should be interpreted with caution when considering their generalizability to other Palestinian refugee camps, schools, or broader student populations.

### 3.3 Research instruments

The World Health Organization (WHO-5) scale for psychological well-being will be translated and used to collect the necessary data from the study sample after adapting it to the Palestinian environment and society. Researchers will use the WHO-5 scale for psychological well-being (39), which has achieved high reliability and validity in measuring psychological well-being. Furthermore, the reliability of the scale will be calculated through a survey sample and will be evaluated to determine internal validity.

The study instrument consists of (5) questions with response weights distributed across six responses: (always, most of the time, a little more than half the time, a little less than half the time, a little of the time, never), with the highest response score (5) representing the best possible psychological well-being (good condition), and the lowest response score (0) representing the worst possible psychological well-being (good condition). Participants who obtain a total score >13 indicate that they enjoy good psychological well-being (good condition).

The study adopted the following tools: a) individual interviews with a group of students and focus groups to examine the extent of their psychological well-being; b) individual interviews with educational counselors and teachers in both schools; c) interviews with a random sample of parents in both schools.

### 3.4 Data analysis

Quantitative data were analyzed using the Statistical Package for the Social Sciences (SPSS). Descriptive statistics, including means and standard deviations, were calculated for all study variables. Paired-samples and independent-samples t-tests were used to examine differences between pre-test and post-test measurements within and between groups. In addition, two-way Analysis of Covariance (ANCOVA) was conducted to examine the effects of group (experimental vs. control), gender (male vs. female), and their interaction on post-test social-emotional skills and psychological well-being outcomes while controlling for baseline scores. Effect sizes were calculated using Cohen’s d and Partial Eta² to assess the practical significance of the findings. Statistical significance was established at p <.05.

Before conducting the statistical analyses, the assumptions underlying ANCOVA were assessed, including normality of distributions, homogeneity of variance, and linear relationships between the covariates and dependent variables. The assumptions were found to be adequately met, supporting the appropriateness of ANCOVA for the present analyses. Given the balanced distribution of participants across groups and the moderate sample size, ANCOVA was deemed an appropriate analytical procedure for evaluating intervention effects.

Qualitative feedback obtained through semi-structured interviews with students, teachers, counselors, parents, and the school principal was reviewed and used to support the interpretation of the quantitative findings and assess the perceived usefulness and acceptability of the intervention. These data served as supplementary evaluative information and were not analyzed as an independent qualitative dataset.

#### 3.4.1 Training program

##### 3.4.1.1 First: objectives of the training program

1. Enhancing social and emotional skills as follows: a) Recognizing, releasing, expressing, and sharing emotions. b) Good listening skills. c) Self-control skills. d) Dealing with stress, peer pressure, conflicts, frustration, fighting, and violence. e) Communication skills. f) Problem-solving skills. g) Understanding and appreciating others and recognizing differences. h) Self-identification and expression skills. i) Personal goal-setting skills. j) Constructive criticism skills. k) Feeling proud of oneself and one’s family and recognizing family differences. l) Adherence to established beliefs and others.
2. Mental health, psychological well-being, and improved well-being among students.
3. Improved social relationships among students and between students and their teachers and parents.

##### 3.4.1.2 Second: training program description

The SET program was designed to enhance protective factors at the individual and group levels, based on social learning theory.

Although the SET program was developed and implemented in an environment that does not resemble the Palestinian environment, the Bible Society, in cooperation with the Palestinian Ministry of Education, adopted the guide (https://palsawa.com/post/339477/) and adapted and modified the guide’s activities to suit the Palestinian context, which suffers from difficult conditions such as poverty, occupation, marginalization, and refugee status. Specifically, the training manual addresses the following sub-skills: a) Recognizing, releasing, expressing, and sharing emotions. b) Good listening skills. c) Self-control skills. e) Dealing with stress, peer pressure, conflicts, frustration, fighting, and violence. f) Communication skills. g) Problem-solving skills. h) Understanding and appreciating others and recognizing differences. i) Self-knowledge and self-expression skills. j) Personal goal-setting skills. k) Constructive criticism skills. l) Feeling proud of oneself and one’s family and recognizing family differences. m) Adherence to established beliefs and others.

The SET program includes more than one skill in each exercise or activity, and often the different skills overlap and blend together; for example, social competence often involves empathy, while self-awareness includes managing emotions. School students will acquire the aforementioned social and emotional skills through interactive learning strategies based on active learning, learning by doing, role-playing, and other methods, far removed from traditional or authoritarian methods based on preaching and traditional teaching. Despite the many social and emotional skills to be implemented, the time allocated for them is carefully considered and has been implemented in numerous public schools across the country, achieving positive results. This has prompted the Ministry to expand the training of counselors to other governorates.

Before the program’s implementation, parents will be invited to a meeting with the research team and trainers to brief them on the project’s objectives, outcomes, requirements, implementation method, and content. The goal is to keep parents informed of the program, which will enhance communication with the program’s implementers at school and help parents strengthen their children’s targeted skills. The weekly meetings will also address any challenges that arise due to the ongoing program evaluation. Parents may also be invited to attend some of the sessions held within schools, whenever possible.

##### 3.4.1.3 Third: program implementation mechanism

*a)* The training program will be implemented for one hour per seventh-grade class in the Jalazone Elementary Schools for Boys and Girls, throughout the first semester, which begins in September and ends approximately mid-January. This translates to an average of 16 training sessions (one hour) for each of the above classes. b) The training will be implemented by Professor Morris in collaboration with members of the research team, as well as with research and teaching assistants and the Bible Society. *c)* Weekly sessions will be held between program implementers, training supervisors, and members of the research team to discuss and review the difficulties and challenges faced by students during the implementation process and provide recommendations. *d)* The school’s psychological and educational counselor will be present to assist in the implementation process and intervene when necessary.

#### 3.4.2 Intervention protocol

Prior implementing the intervention in schools, the research team participated in a structured capacity-building process designed to ensure methodological rigor and fidelity in delivering the SET (Social and Emotional Training) program. This training consisted of three consecutive workshops organized by the Palestinian Bible Society. The first workshop, held at the Society’s headquarters in Ramallah on 7 October 2023, introduced the core theoretical foundations of social and emotional learning (SEL), the conceptual framework of psychological well-being, and the overall structure and aims of the SET manual.

The second workshop, conducted on 21 October 2023 at Carmel Hotel, focused on developing practical facilitation skills. It included guided simulations of classroom activities and training on strategies for managing individual and group differences within diverse learning environments. The third and final workshop took place at Birzeit University on 28 October 2023 and involved field-based simulations and readiness assessments to ensure that all researchers were fully prepared to deliver the program in a standardized and consistent manner across schools in Al-Jalazone Refugee Camp.

Upon completion of the training, the intervention was implemented with Grade 7 students in both UNRWA (MALE AND FAMLE) schools in the camp. The SET program consisted of a structured series of experiential activities aimed at strengthening key SEL competencies, including self-awareness, emotion regulation, empathy, valuing diversity, effective communication, and psychological resilience. Fidelity to the intervention protocol was monitored through classroom observation checklists, facilitator reflection logs, and pre- and post-intervention assessments of students’ psychological well-being.

#### 3.4.3 Program rationale

The choice of the SET program was guided by a combination of theoretical, contextual, and methodological considerations that align with international best practices in SEL intervention research. SET provides a comprehensive, evidence-informed framework that directly targets the essential components of psychological well-being among adolescents—particularly those living in socioeconomically and politically vulnerable environments.

The international literature consistently highlights the need for SEL programs that foster emotional regulation, interpersonal skills, and resilience, especially among youth living in refugee camps who are frequently exposed to chronic stress, instability, and limited psychosocial resources. SET addresses these needs by offering structured, developmentally appropriate activities that enhance students’ emotional and social competencies. Additionally, SET is highly feasible for implementation in under-resourced educational settings, as its activities require minimal materials and can be delivered within existing classroom structures. The availability of formal, standardized training provided by the Palestinian Bible Society further strengthened the methodological reliability of the program, ensuring consistent delivery by all facilitators. Collectively, these factors made SET the most appropriate and scientifically defensible intervention for this study.

#### 3.4.4 Alignment with global SEL frameworks

The SET program aligns closely with internationally recognized SEL models, particularly the CASEL framework of five core competencies—self-awareness, self-management, social awareness, relationship skills, and responsible decision-making. Through experiential learning activities such as role-plays, guided reflections, cooperative group tasks, and emotional literacy exercises, the program translates these competencies into culturally relevant and developmentally suitable practices. Furthermore, SET is consistent with WHO guidelines on psychosocial and emotional support for school-aged children, emphasizing inclusive learning environments, gender-sensitive approaches, and strategies that bolster mental and emotional resilience. This alignment with global SEL frameworks enhances the scientific credibility of the intervention and positions the study within the broader international discourse on effective SEL programming.

#### 3.4.5 Cultural adaptation of the SET program in the Palestinian context

Although grounded in universal SEL principles, the SET program used in this study underwent deliberate cultural adaptation to ensure its relevance and resonance within the Palestinian context. Adaptations were implemented across several dimensions. Linguistically, activities and reflective prompts were translated and reframed using terminology familiar and meaningful to students in UNRWA and public schools. Socially, the program incorporated themes that reflect the everyday realities of Palestinian children, including community cohesion, coping with collective stress, navigating mobility restrictions, and valuing diversity within the camp environment.

In addition, activities were modified to align with local gender norms, classroom dynamics, and the psychosocial challenges associated with camp life, such as overcrowding, family strain, and exposure to political instability. These cultural adaptations ensured that the program was not only applicable but also emotionally relevant and contextually authentic for the target population. Consequently, the intervention was better positioned to produce meaningful improvements in students’ psychological well-being and social-emotional competencies.

## 4 Findings and discussion

### 4.1 Hypotheses testing

#### 4.1.1 Hypothesis 1

There are no statistically significant differences between the mean scores of female students in the experimental and control groups in the pre-test and post-test measurements on the social-emotional skills and psychological well-being scale.

**Figure 1:**
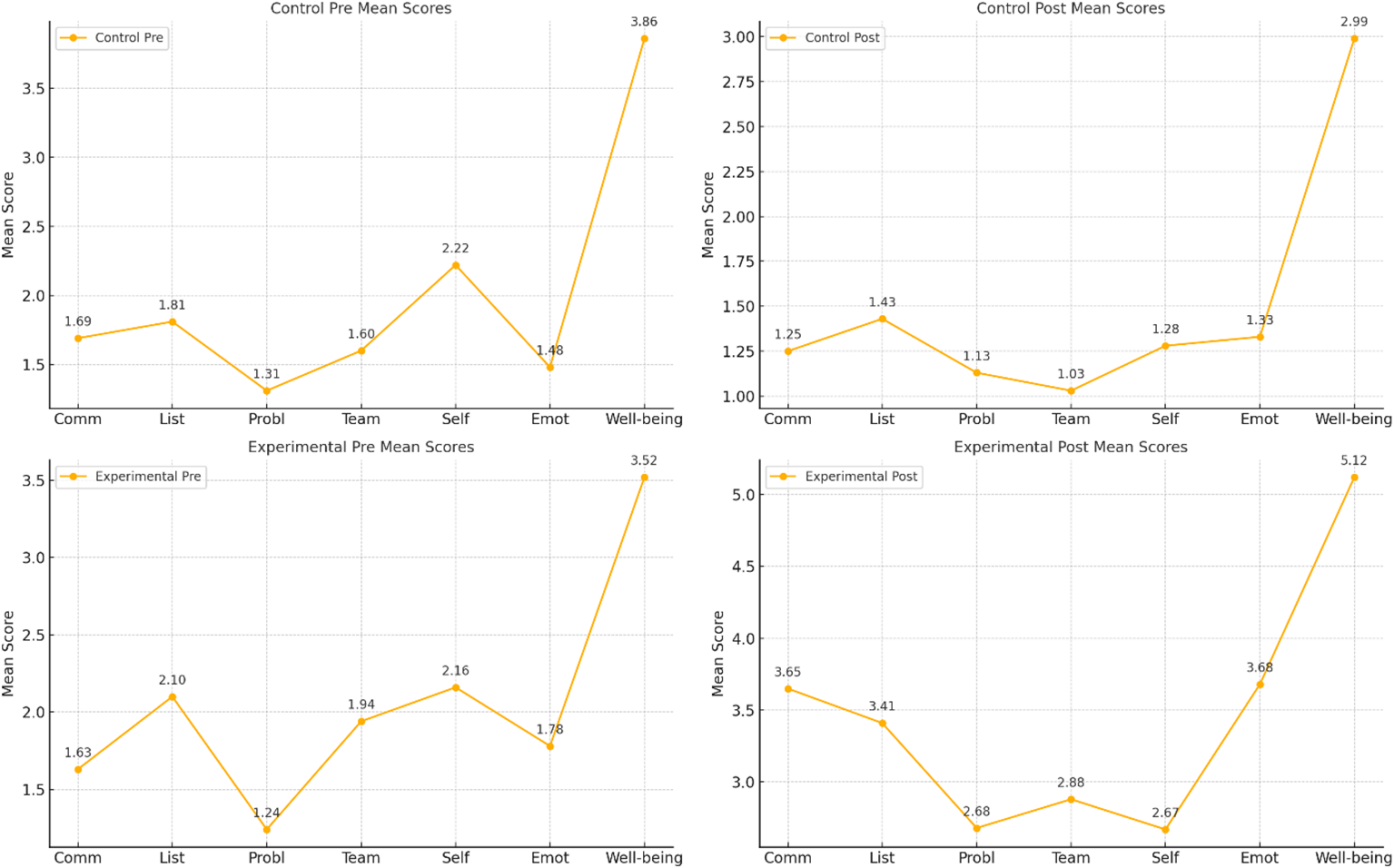
Pre- and post-test mean scores of the control and experimental groups on the social-emotional skills and psychological well-being scale. Source(s): Authors’ own work.

As shown in **Table 1** and **Figure 1**, the pre-test scores revealed no statistically significant differences between the control and experimental groups across most social-emotional skills and psychological well-being measures, confirming the baseline equivalence of the groups. Following the implementation of the proposed program, the experimental group exhibited substantial improvements in all assessed variables, including communication (COMM), listening (LIST), problem-solving (PROBL), teamwork (TEAM), self-awareness (SELF), emotional regulation (EMOT), and overall psychological well-being (WELL). In contrast, the control group displayed only minor changes from pre-test to post-test. Furthermore, effect size estimates (Cohen’s d) indicate that the observed post-test differences were not only statistically significant but also practically meaningful, underscoring the effectiveness of the intervention in enhancing social-emotional competencies and psychological well-being among female students.

**Table 1:** Pre-test and post-test mean scores (M ± SD) of control and experimental female students on social-emotional skills and psychological well-being, with t-values, significance levels, and effect sizes (Cohen’s d).

Variables	Measurement	Control females (N = 30) M ± SD	t	Sig.	Cohen’s d	Experimental females (N = 35) M ± SD	t	Sig.	Cohen’s d
Comm	Pre	1.69 ± 0.47	3.85	0.001	0.70	1.63 ± 0.53	-18.09	0.000	3.06-
	Post	1.25 ± 0.35				3.65 ± 0.34			
List	Pre	1.81 ± 0.72	1.93	0.064	0.35	2.10 ± 1.04	-5.78	0.000	0.98-
	Post	1.43 ± 0.62				3.41 ± 0.70			
Probl	Pre	1.31 ± 0.71	1.78	0.086	0.32	1.24 ± 0.46	-6.48	0.000	1.10-
	Post	1.13 ± 0.34				2.68 ± 1.31			
Team	Pre	1.60 ± 0.72	4.01	0.000	0.73	1.94 ± 1.13	-2.81	0.008	0.48-
	Post	1.03 ± 0.18				2.88 ± 1.34			
Self	Pre	2.22 ± 0.73	6.39	0.000	1.16	2.16 ± 0.99	-3.40	0.002	0.58-
	Post	1.28 ± 0.35				2.67 ± 0.60			
Emot	Pre	1.48 ± 0.51	1.34	0.191	0.24	1.78 ± 0.89	-11.59	0.000	1.96-
	Post	1.33 ± 0.44				3.68 ± 0.43			
Well-being	Pre	3.86 ± 1.20	3.24	0.003	0.59	3.52 ± 0.99	-4.85	0.000	1.11-
	Post	2.99 ± 0.69				5.12 ± 0.99			

Comm, Communication; List, Listening; Probl, Problem Solving; Team, Teamwork; Self, Self-Awareness; Emot, Emotional Regulation; M, Mean; SD, Standard Deviation; Sig., p-value; Cohen’s d, Effect Size.

Source(s): Authors’ own work.

Based on the statistically significant improvements observed across all measured variables, the null hypothesis was rejected, indicating that the SET program had a significant positive effect on female students’ social-emotional skills and psychological well-being.

This figure illustrates the mean scores of female students in the control and experimental groups on the pre-test and post-test across the components of the social-emotional skills and psychological well-being scale. Each subplot separately highlights one testing condition to provide clear insight into the progression within each group.

**Figure 2:**
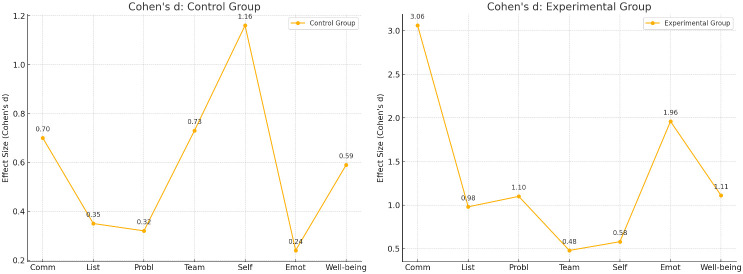
Effect sizes (Cohen’s d) for the control and experimental groups across the social-emotional and psychological well-being variables. Source(s): Authors’ own work.

**Figure 2** displays the effect sizes (Cohen’s d) for the control and experimental groups across the measured variables. Larger effect sizes observed in the experimental group indicate a stronger impact of the intervention, particularly on communication, emotional regulation, and psychological well-being.

#### 4.1.2 Hypothesis 2

There are no statistically significant differences between the mean scores of male students in the experimental and control groups in the pre-test and post-test measurements on the social-emotional skills and psychological well-being scale.

As shown in **Table 2** and **Figure 2**, the pre-test scores demonstrated no statistically significant differences between the control and experimental groups across most social-emotional skills and psychological well-being domains, confirming the initial baseline equivalence between male students in both groups. After the implementation of the proposed program, however, the experimental group exhibited marked improvements in all assessed variables, including communication (COMM), listening (LIST), problem-solving (PROBL), teamwork (TEAM), self-awareness (SELF), emotional regulation (EMOT), and overall psychological well-being (WELL).

**Table 2:** Pre-test and post-test mean scores (M ± SD) of control and experimental male students on social-emotional skills and psychological well-being, with t-values, significance levels, and effect sizes (Cohen’s d).

Variables	Measurement	Control males (N = 34) M ± SD	t	Sig.	Cohen’s d	Experimental males (N = 36) M ± SD	t	Sig.	Cohen’s d
Comm	Pre	1.63 ± 0.34	3.18	0.003	0.55	1.72 ± 0.31	-10.69	0.000	1.78-
	Post	1.26 ± 0.49				3.00 ± 0.48			
List	Pre	1.98 ± 0.57	3.45	0.002	0.59	2.01 ± 0.47	-2.84	0.007	0.47-
	Post	1.48 ± 0.48				2.61 ± 1.05			
Probl	Pre	1.19 ± 0.37	1.23	0.226	0.21	1.23 ± 0.47	-3.56	0.001	0.59-
	Post	1.10 ± 0.29				1.83 ± 1.07			
Team	Pre	1.20 ± 0.54	1.01	0.325	0.17	1.36 ± 0.68	-2.57	0.014	0.43-
	Post	1.09 ± 0.38				2.08 ± 1.31			
Self	Pre	2.08 ± 0.59	5.09	0.000	0.87	2.27 ± 0.43	-16.74	0.000	2.79-
	Post	1.49 ± 0.25				3.53 ± 0.27			
Emot	Pre	2.14 ± 0.67	4.73	0.000	0.81	1.96 ± 0.72	-8.68	0.000	1.45-
	Post	1.52 ± 0.38				3.01 ± 0.66			
Well-being	Pre	3.47 ± 1.18	2.33	0.026	0.40	3.46 ± 0.78	-2.71	0.010	0.45-
	Post	2.80 ± 0.98				4.10 ± 0.97			

Comm, Communication; List, Listening; Probl, Problem Solving; Team, Teamwork; Self, Self-Awareness; Emot, Emotional Regulation; M, Mean; SD, Standard Deviation; Sig., p-value; Cohen’s d, Effect Size.

Source(s): Authors’ own work.

In contrast, the control group of male students showed only slight and statistically limited changes from pre-test to post-test. Notably, the effect size estimates (Cohen’s d) revealed big and practically meaningful differences, particularly in self-awareness (d = 2.79), communication (d = 1.78), and emotional regulation (d = 1.45). These findings highlight the effectiveness of the intervention program in enhancing social-emotional competencies and psychological well-being among male students, with large and substantial gains observed across the majority of skills assessed.

Accordingly, the null hypothesis was rejected, providing further evidence that the intervention significantly enhanced social-emotional competencies and psychological well-being among male students.

**Figure 3:**
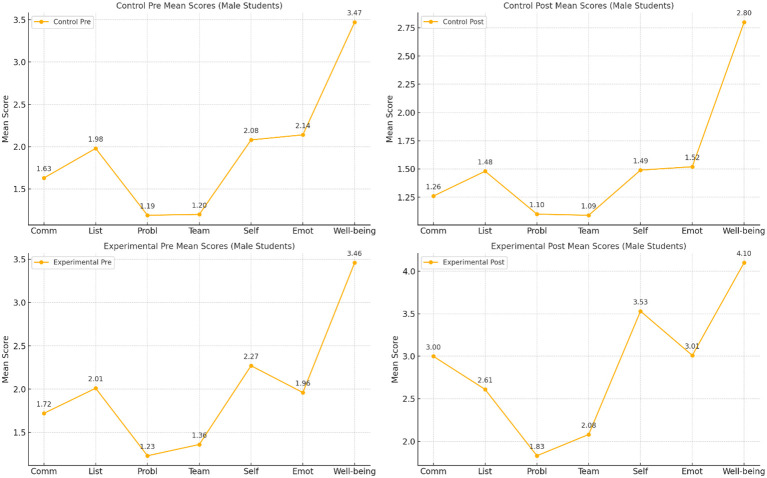
Pre- and post-test mean scores of the control and experimental groups (male students) on the social-emotional skills and psychological well-being scale. Source(s): Authors’ own work.

**Figure 3** illustrates the pre-test and post-test mean scores of male students in both control and experimental groups across all variables of the social-emotional skills and psychological well-being scale. The subplots allow a clear visual comparison of score development within each group.

**Figure 4:**
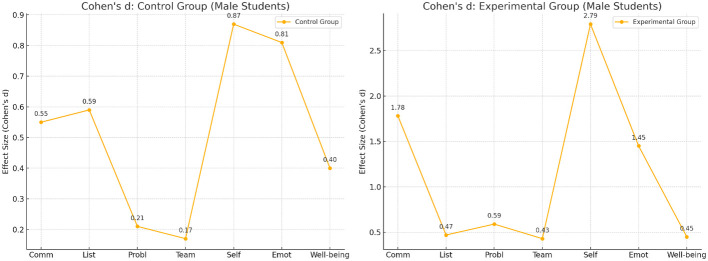
Effect sizes (Cohen’s d) for the control and experimental groups (male students) across the social-emotional and psychological well-being variables. Source(s): Authors’ own work.

**Figure 4** presents the effect sizes (Cohen’s d) for male students in the control and experimental groups across all measured variables. Higher effect sizes in the experimental group reflect the significant impact of the intervention, especially on self-awareness, emotional regulation, and communication skills.

#### 4.1.3 Hypothesis 3

There are no statistically significant differences in the post-test means of social-emotional skills and psychological well-being variables (COMM, LIST, PROB, TEAM, SELF, EMOT, WELL-BEING) attributable to group (experimental vs. control), gender (male vs. female), or their interaction.

The results of the analysis of variance in **Table 3** above indicate that the proposed training program produced statistically significant differences between the groups, with the Group factor emerging as the most influential across all dependent variables. The very high values of Partial Eta² (e.g., η² = 0.866 for communication [COMM] and η² = 0.838 for self-concept [SELF]) confirm a large effect size, underscoring the strong impact of the intervention on enhancing social-emotional skills and psychological well-being.

**Table 3:** Two-way ANCOVA results with partial Eta² values for social-emotional skills and psychological well-being variables.

Variables	Source of variation	Sum of squares	df	Mean square	F	Sig.	Partial Eta²
COMMUPOST	(COMM Pre-test)	1.488	1	1.488	8.679	0.004	0.063
	Group	143.962	1	143.962	839.908	0.000	0.866
	Gender	3.341	1	3.341	19.491	0.000	0.13
	Group × Gender	3.2	1	3.2	18.669	0.000	0.126
	Error	22.282	130	0.171			
	Total	174.233	135				
LISTNGPOST	(LISTN Pre-test)	3.030	1	3.030	5.481	0.021	0.040
	Group	83.485	1	83.485	150.995	0.000	0.537
	Gender	4.522	1	4.522	8.178	0.005	0.059
	Group × Gender	6.868	1	6.868	12.422	0.001	0.087
	Error	71.877	130	0.553			
	Total	166.859	135				
PROBPPOST	(PROBP Pre-test)	6.718	1	6.718	8.85	0.003	0.064
	Group	44.239	1	44.239	58.279	0.000	0.31
	Gender	5.679	1	5.679	7.481	0.007	0.054
	Group × Gender	6.401	1	6.401	8.433	0.004	0.061
	Error	98.682	130	0.759			
	Total	161.770	135				
TEAPOST	(TEA Pre-test)	8.285	1	8.285	8.997	0.003	0.065
	Group	73.911	1	73.911	80.264	0.000	0.382
	Gender	8.497	1	8.497	9.227	0.003	0.066
	Group × Gender	7.019	1	7.019	7.622	0.007	0.055
	Error	119.710	130	0.921			
	Total	206.993	135				
SELFPOST1	(SELF Pre-test)	1.457	1	1.457	9.969	0.002	0.071
	Group	98.087	1	98.087	671.284	0.000	0.838
	Gender	9.763	1	9.763	66.819	0.000	0.339
	Group × Gender	3.18	1	3.18	21.766	0.000	0.143
	Error	18.995	130	0.146			
	Total	133.962	135				
EMONOST12	(EMON Pre-test)	1.511	1	1.511	6.399	0.013	0.047
	Group	122.077	1	122.077	517.008	0.000	0.799
	Gender	2.819	1	2.819	11.939	0.001	0.084
	Group × Gender	4.98	1	4.98	21.092	0.000	0.14
	Error	30.696	130	0.236			
	Total	162.982	135				
WELLPOST12333	(WELL Pre-test)	4.108	1	4.108	5.468	0.021	0.040
	Group	94.624	1	94.624	125.950	0.000	0.492
	Gender	13.774	1	13.774	18.334	0.000	0.124
	Group × Gender	4.972	1	4.972	6.618	0.011	0.048
	Error	97.667	130	0.751			
	Total	219.509	135				

Comm, Communication; List, Listening; Probl, Problem Solving; Team, Teamwork; Self, Self-Awareness; Emot, Emotional Regulation; M, Mean; SD, Standard Deviation; Sig., p-value; Partial Eta², Effect Size.

Source(s): Authors’ own work.

The descriptive means further substantiate these findings as illustrated in **Table 4**. The experimental females consistently achieved the highest post-test scores across all variables—for instance, COMM (M = 3.65) compared to the control females (M = 1.25), and WELL (M = 5.12) compared to the control females (M = 2.99). The experimental males also outperformed their control counterparts across all measures, although the gains were generally more pronounced among females, except in self-concept (SELF), where the experimental males (M = 3.54) exceeded the experimental females (M = 2.67).

**Table 4:** Descriptive statistics (means) of the experimental and control groups (males and females) on the post-test for social-emotional skills and psychological well-being variables.

Variables	Experimental males	Experimental females	Control males	Control females
COMM	3.000	3.6484	1.2624	1.2538
LIST	2.6111	3.4143	1.4853	1.4333
PROB	1.8333	2.6857	1.1029	1.1333
TEAM	2.0833	2.8857	1.0882	1.0333
SELF	3.5389	2.6743	1.4912	1.2817
EMOT	3.0159	3.6816	1.5252	1.3381
WELL-BEING	4.100	5.120	2.800	2.9933

COMM, Communication; LIST, Listening; PROB, Problem Solving; TEAM, Teamwork; SELF, Self-Awareness; EMOT, Emotional Regulation.

Source(s): Authors’ own work.

The Gender factor also reached statistical significance in several variables, with effect sizes ranging from medium to large (η² = 0.124–0.339), indicating differential responses between males and females. The Group × Gender interaction was significant in multiple domains (e.g., LIST, SELF, EMO), with medium effect sizes (η² ≈ 0.055–0.143), suggesting that the program’s impact was not homogeneous across gender. In contrast, the Pre-test covariate exhibited limited influence (η² ≈ 0.04–0.07), supporting the assumption of baseline equivalence among groups.

Overall, the findings provide robust evidence for the effectiveness of the intervention in improving communication, listening, problem-solving, teamwork, self-concept, emotional regulation, and psychological well-being. They also highlight the importance of considering gender differences in program design and implementation. Because statistically significant effects were observed for the group factor, gender factor, and several group-by-gender interactions (p <.05), the null hypothesis was rejected. These results indicate that post-test outcomes differed significantly according to group membership, gender, and, in several cases, the interaction between the two factors.

This work represents a significant contribution to the field of educational and mental health interventions in Palestinian school settings, providing a foundation for more inclusive and evidence-based approaches to promoting social-emotional development and psychological well-being among adolescents.

**Figure 5:**
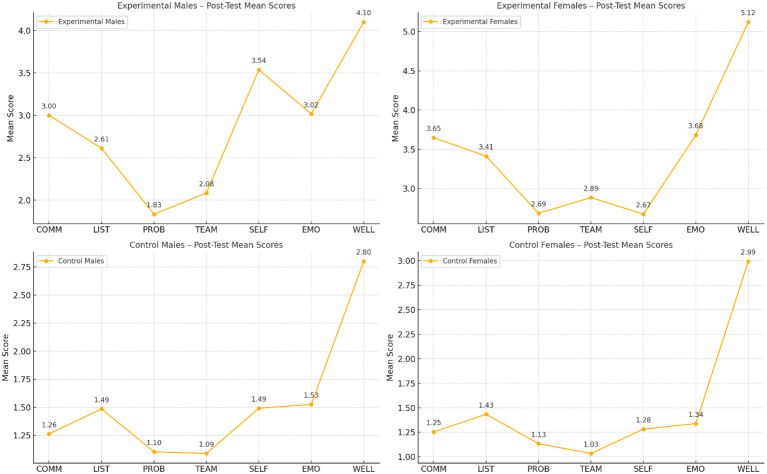
Post-test mean scores by group and gender across social-emotional and psychological well-being variables (separate plots). Source(s): Authors’ own work.

**Figure 5** separately illustrates the post-test mean scores for each group—experimental males, experimental females, control males, and control females—across the seven variables of the social-emotional and psychological well-being scale. The breakdown highlights within-group trends, supporting the interpretation of ANCOVA results.

**Figure 6:**
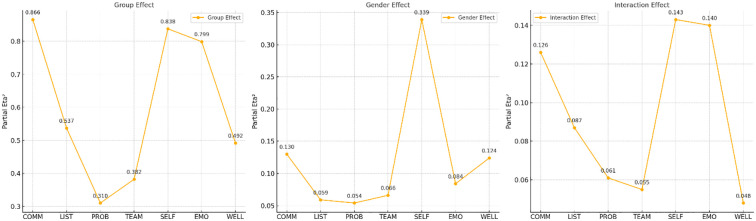
Partial Eta² values for the effects of group, gender, and their interaction on post-test outcomes. Source(s): Authors’ own work.

**Figure 6** presents the partial eta-squared values (η²) separately for the group effect, gender effect, and their interaction across all measured variables. Each subplot highlights the variance explained by each source of influence, supporting the findings from the two-way ANCOVA analysis.

**Figure 6** presents a clear visual comparison of the partial eta-squared (η²) values derived from the two-way ANCOVA, highlighting the effect sizes associated with group membership (experimental vs. control), gender (male vs. female), and their interaction across all measured variables. The group effect demonstrated consistently large effect sizes, particularly for communication (COMM = 0.866), self-awareness (SELF = 0.838), and emotional regulation (EMO = 0.799). These values substantially exceed the conventional threshold for a large effect (η² ≥ 0.14), indicating that the experimental intervention had a strong and meaningful impact on students’ social-emotional skills and psychological well-being. The gender effect was also notable, with moderate to large effect sizes observed in self-awareness (SELF = 0.339) and communication (COMM = 0.130), suggesting that female students may have responded differently to the intervention, particularly in domains related to internal awareness and interpersonal expression. Furthermore, the interaction effect between group and gender showed meaningful contributions in several variables, including self-awareness (SELF = 0.143), communication (COMM = 0.126), and emotional regulation (EMO = 0.140). These findings imply that the effectiveness of the intervention was not uniform across genders and that males and females exhibited different patterns of change depending on their group membership.

## 5 Conclusion and policy implications

### 5.1 Policy implications

This study has significant implications for educational policy and practice. One of the key programs that provides students with essential competencies for effective social interaction throughout childhood and adolescence is the Social and Emotional Training (SET) program. Through participation in the program, students learn how to manage their own emotions, understand the emotions of others, resolve conflicts constructively, and develop healthy coping and life-management skills.

The findings of the present study suggest that structured social-emotional learning interventions can serve as effective tools for enhancing psychological well-being among adolescents exposed to conflict-related stressors and challenging living conditions.

These findings are consistent with previous research highlighting the psychological vulnerabilities experienced by Palestinian refugee children and adolescents. Wilson et al. (18) reported that Palestinian refugee children are at increased risk of mental health difficulties and psychosomatic symptoms, while Nathani et al. (8) found that mental health problems among refugee adolescents were associated with loneliness, anxiety, and school absenteeism. Similarly, Al Issa (19) documented the multiple forms of trauma experienced by Palestinian refugee children and emphasized the importance of protective factors such as self-efficacy and social support. At the international level, Douglas et al. (20) demonstrated that exposure to displacement, conflict, and traumatic events substantially increases the risk of anxiety, depression, and other mental health disorders. Collectively, these findings reinforce the need for preventive interventions that strengthen resilience and psychological well-being among vulnerable student populations.

Teachers, counselors, and UNRWA’s psychological and educational counseling departments may benefit from the findings of this study, as they provide evidence-based guidance for supporting students’ social-emotional development and psychological well-being.

This study implemented a Social and Emotional Training (SET) program among seventh-grade students in boys’ and girls’ schools within Jalazone Refugee Camp. The program focused on preventing the deterioration of mental health and psychological well-being while strengthening students’ social and emotional competencies and encouraging the practical application of these skills in their daily lives.

Furthermore, the program was adapted to reflect the social, cultural, and educational characteristics of the Palestinian context, which may enhance its relevance and applicability in similar educational settings.

The positive outcomes observed in the current study are also consistent with previous evaluations of Social and Emotional Training (SET) programs. Kimber, Sandell, and Bremberg (30) reported improvements in social competence, self-awareness, empathy, motivation, and reductions in bullying behaviors among students participating in SET interventions. Likewise, Kimber (28) found that SET programs contributed to the prevention of mental health problems, improved school satisfaction, and reduced school dropout rates. Sandell and Kimber (23) further demonstrated that social-emotional learning interventions can positively influence students’ socio-emotional development, particularly among high-risk groups.

More recent international evidence further strengthens these conclusions. Lee and Yoo (31), in a systematic review of SEL interventions, reported that such programs improve adolescents’ emotion regulation, happiness, and overall mental well-being. Similarly, de Mooij et al. (32) found that SEL interventions positively influenced school climate and psychosocial adjustment, whereas Belaire et al. (33) demonstrated significant associations between social-emotional learning, resilience, and psychological well-being among school-aged children. The consistency between these international findings and the present results provides additional support for the effectiveness of SEL-based interventions across diverse educational and cultural contexts.

From a policy perspective, the findings suggest that the Palestinian Ministry of Education and UNRWA may benefit from integrating social and emotional learning programs into school curricula and student support services.

For UNRWA specifically, the findings support the incorporation of structured SET-based activities within existing psychosocial support, counseling, and student well-being programs. Such integration may strengthen emotional regulation, resilience, interpersonal competencies, and psychological well-being among refugee adolescents who are regularly exposed to chronic stressors and challenging living conditions.

Given the psychological challenges faced by students living in refugee camps and conflict-affected environments, preventive and developmental interventions should be considered an essential component of educational practice rather than supplementary activities.

The incorporation of SET principles within school counseling programs, classroom activities, and extracurricular initiatives may contribute to promoting positive mental health, emotional regulation, social competence, and adaptive coping skills among students.

The proposed interventions focus on strengthening protective factors and resilience rather than solely addressing psychological symptoms or behavioral problems. Through the SET program, students acquire practical skills related to emotional regulation, problem-solving, empathy, communication, cooperation, and conflict resolution.

Additional research on the psychological well-being of Palestinian students remains necessary. A comprehensive understanding of the psychological needs of this population can contribute to the development of more effective mental health promotion and prevention programs.

Research has consistently demonstrated that community-based and school-based interventions represent effective approaches for supporting mental health and well-being in conflict-affected populations. Within schools, such interventions provide a unique opportunity for the early identification of psychological difficulties and the delivery of accessible support services to students.

Consequently, policymakers should consider expanding school-based mental health initiatives and increasing investment in preventive programs that strengthen students’ resilience, social competence, and emotional well-being.

Previous research has shown that school counseling programs can significantly reduce psychological symptoms and improve psychological well-being among Palestinian children and adolescents. Integrating accessible and sustainable counseling services within schools may facilitate the implementation of evidence-based practices that promote positive mental health outcomes.

The higher scores observed among female students in several social-emotional domains may be explained by developmental and socialization factors. Previous research suggests that adolescent girls tend to demonstrate greater emotional awareness, interpersonal sensitivity, empathy, and willingness to express emotions compared with boys. These characteristics may have enabled female students to engage more actively with the SET program activities, particularly those related to communication, emotional regulation, and interpersonal relationships. Consequently, female participants may have derived greater benefits from specific components of the intervention designed to strengthen social-emotional competencies and psychological well-being.

These findings are broadly consistent with previous research suggesting that female adolescents often report higher emotional awareness and different patterns of psychological distress than males (8, 17). Therefore, gender-sensitive school-based interventions and future studies are recommended to better address the specific mental health and well-being needs of both male and female students.

Furthermore, the successful implementation of SEL programs depends heavily on teacher preparation and professional development. Consistent with Kimber, Skoog, and Sandell (29), the current findings suggest that teachers play a critical role in fostering students’ social-emotional development and psychological well-being. Therefore, educational authorities and UNRWA should consider incorporating SEL competencies into both pre-service teacher education and in-service professional development programs to ensure the sustainable and effective implementation of social-emotional learning initiatives.

Finally, because the current study was conducted within a refugee camp setting, its findings support the development of comprehensive school- and community-based approaches that promote resilience, psychological well-being, and social-emotional competence among refugee adolescents. Such initiatives may contribute not only to improving students’ mental health outcomes but also to enhancing their educational engagement, social adjustment, and overall quality of life.

### 5.2 Limitations and future research directions

This study has several limitations that should be acknowledged. First, although the findings support the effectiveness of the Social and Emotional Training (SET) program in enhancing psychological well-being, further research is needed to identify the specific program components that contribute most strongly to positive outcomes. Such evidence could facilitate the development of more targeted and efficient interventions.

Second, additional studies are needed to strengthen the translation of evidence-based social and emotional learning (SEL) practices into routine school settings, particularly in resource-constrained and conflict-affected environments. Future research should explore scalable and sustainable implementation strategies that support the integration of SEL interventions into everyday educational practice.

Third, educational policies and school practices should continue to incorporate evidence-based SEL interventions that promote positive youth development, resilience, and psychological well-being. Future studies may examine how different implementation models influence program effectiveness across diverse educational contexts.

Fourth, future research should investigate whether individualized or adaptive intervention models can further improve student outcomes. Identifying the most effective elements of SEL programs may allow practitioners to tailor intervention content and delivery according to the needs of specific student populations and school environments.

Finally, the study sample was purposively selected from a single UNRWA refugee camp (Jalazone Camp), which may limit the generalizability of the findings to other refugee camps, schools, or Palestinian student populations. In addition, gender-related findings should be interpreted cautiously, as gender roles and expectations are influenced by local cultural and social contexts. Furthermore, the questionnaire did not distinguish between biological sex and gender identity, which may have limited a more nuanced understanding of gender-related differences. Future studies should employ larger and more diverse samples and examine these issues across different geographical and cultural settings.

### 5.3 Conclusion

This study aims to investigate the efficacy of a suggested program to improve social and emotional skills in fostering mental health in seventh-grade children at the United Nations Relief and Works Agency for Palestine Refugees (UNRWA)-run Jalazone Basic School for Boys and Girls.

This study indicated a link between the influence of social and emotional skills and the reported mental health of Palestinian children in UNRWA schools across both genders. The influence of these abilities on enhancing mental health and well-being was found to be high, and the deterioration in mental health and well-being may have been aggravated by sociopolitical settings as well as insufficient intervention resources.

Psychological symptoms associated with the development of these abilities differed across male and female pupils and across locations, prompting more research into gender disparities among Palestinian adolescents. More thorough research addressing wider and more influential variables, while accounting for sample size, is required to design school-based and gender-sensitive treatments for Palestinian kids to better understand and invest in their mental health.

The pre-test findings confirmed the groups’ baseline equivalency by showing no statistically significant differences between the experimental and control groups on the majority of social-emotional skills and psychological well-being measures. Following the proposed program’s implementation, the experimental group demonstrated significant improvements in all evaluated variables, such as listening (LIST), communication (COMM), problem-solving (PROBL), teamwork (TEAM), self-awareness (SELF), emotional regulation (EMOT), and overall psychological well-being (WELL).

The pre-test results confirmed the original baseline equivalency between male students in both groups, showing no statistically significant differences between the experimental and control groups across the majority of social-emotional skills and psychological well-being categories. However, the experimental group demonstrated significant gains in all evaluated variables following the implementation of the suggested program, including listening (LIST), communication (COMM), problem-solving (PROBL), teamwork (TEAM), self-awareness (SELF), emotional regulation (EMOT), and overall psychological well-being (WELL).

According to the findings of the Two-Way ANCOVA analysis of variance, the suggested training regimen resulted in statistically significant group differences, with the Group factor having the greatest influence on all dependent variables. Consequently, the null hypothesis is disproved, and the first alternative hypothesis is accepted. As a result, there are notable variations in the evaluations of post-communication skills among male students, and these variations benefit the experimental group.

As a result, there are notable variations in the evaluations of post-communication skills among male students, and these variations benefit the experimental group. Additionally, the investigation indicated that the null hypothesis is accepted and the first alternative hypothesis is rejected. Consequently, the typical male students’ pre- and post-communication ability assessments do not differ significantly among themselves.

This study fills a knowledge gap in gender differences in emotional and social skills and their significance in promoting psychological well-being among UNRWA students in the Jalazone Palestinian refugee camp. Addressing these variations among this student population and understanding their underlying reasons will aid in developing effective, culturally relevant, and individualized treatments for each group.

## Data Availability

The original contributions presented in the study are included in the article/supplementary material. Further inquiries can be directed to the corresponding author.
